# The Early Fragmentation of a Bovine Dermis-Derived Collagen Barrier Membrane Contributes to Transmembraneous Vascularization—A Possible Paradigm Shift for Guided Bone Regeneration

**DOI:** 10.3390/membranes11030185

**Published:** 2021-03-09

**Authors:** Eleni Kapogianni, Said Alkildani, Milena Radenkovic, Xin Xiong, Rumen Krastev, Ignacio Stöwe, James Bielenstein, Ole Jung, Stevo Najman, Mike Barbeck, Daniel Rothamel

**Affiliations:** 1 Private Practice, 10623 Berlin, Germany; eleni@ekap.de; 2 BerlinAnalytix GmbH, 12109 Berlin, Germany; said.alkildani@berlinanalytix.com (S.A.); james.bielen@gmail.com (J.B.); 3 Scientific Research Center for Biomedicine, Department for Cell and Tissue Engineering, Faculty of Medicine, University of Nis, 18108 Nis, Serbia; milena1390nis@gmail.com; 4 NMI Natural and Medical Sciences Institute at the University of Tübingen, 72770 Reutlingen, Germany; xin.xiong@nmi.de (X.X.); rumen.krastev@reutlingen-university.com (R.K.); 5 Faculty of Applied Chemistry, Reutlingen University, 72762 Reutlingen, Germany; 6 Helios Klinikum Emil von Behring, Gefäßzentrum Berlin Südwest, 14165 Berlin, Germany; ignacio.stoewe@gmx.net; 7 Clinic and Policlinic for Dermatology and Venereology, University Medical Center Rostock, 18057 Rostock, Germany; ole.tiberius.jung@gmail.com; 8 Department of Biology and Human Genetics, Department for Cell and Tissue Engineering, Faculty of Medicine, University of Nis, 18108 Nis, Serbia; stevo.najman@medfak.ni.ac.rs; 9 Department of Ceramic Materials, Chair of Advanced Ceramic Materials, Institute for Materials Science and Technologies, Technical University of Berlin, 10587 Berlin, Germany; 10 Department of Oral and Maxillofacial Plastic Surgery, Evangelic Johanniter Hospital Bethesda Mönchengladbach, 41061 Mönchengladbach, Germany; Daniel.rothamel@mg.johanniter-kliniken.de; 11 Department of Oral and Maxillofacial Plastic Surgery, Heinrich-Heine Universität Düsseldorf, 40225 Düsseldorf, Germany

**Keywords:** tissue source, bovine collagen, porcine collagen, barrier membrane, Guided Bone Regeneration (GBR), tissue regeneration, transmembraneous vascularization, bovine dermis, porcine pericardium

## Abstract

Collagen-based barrier membranes are an essential component in Guided Bone Regeneration (GBR) procedures. They act as cell-occlusive devices that should maintain a micromilieu where bone tissue can grow, which in turn provides a stable bed for prosthetic implantation. However, the standing time of collagen membranes has been a challenging area, as native membranes are often prematurely resorbed. Therefore, consolidation techniques, such as chemical cross-linking, have been used to enhance the structural integrity of the membranes, and by consequence, their standing time. However, these techniques have cytotoxic tendencies and can cause exaggerated inflammation and in turn, premature resorption, and material failures. However, tissues from different extraction sites and animals are variably cross-linked. For the present in vivo study, a new collagen membrane based on bovine dermis was extracted and compared to a commercially available porcine-sourced collagen membrane extracted from the pericardium. The membranes were implanted in Wistar rats for up to 60 days. The analyses included well-established histopathological and histomorphometrical methods, including histochemical and immunohistochemical staining procedures, to detect M1- and M2-macrophages as well as blood vessels. Initially, the results showed that both membranes remained intact up to day 30, while the bovine membrane was fragmented at day 60 with granulation tissue infiltrating the implantation beds. In contrast, the porcine membrane remained stable without signs of material-dependent inflammatory processes. Therefore, the bovine membrane showed a special integration pattern as the fragments were found to be overlapping, providing secondary porosity in combination with a transmembraneous vascularization. Altogether, the bovine membrane showed comparable results to the porcine control group in terms of biocompatibility and standing time. Moreover, blood vessels were found within the bovine membranes, which can potentially serve as an additional functionality of barrier membranes that conventional barrier membranes do not provide.

## 1. Introduction

In dental implantology, the necessity for adequate quantity and quality of bone for implant placement and its stabilization can require a bone augmentation procedure prior to implantation. In this context, Guided Bone Regeneration (GBR) has become one of the most established techniques for jawbone augmentation [1]. GBR is nearly always performed with the use of a barrier membrane to seclude the bone defect from the infiltration of soft tissue and especially epithelial cells that could otherwise invade the defect area and interact with the bone regeneration process [2]. By preventing the ingrowth of these faster growing cells, bone cells can repopulate the bone defect space, and a regenerative environment can be established [3]. Nowadays, most often, resorbable membranes are used for most GBR procedures, as their application does not require a second surgery for their extraction as in the case of nonresorbable membranes [3]. In this context, collagen was and is still used as a base material due to the belief that collagen—from every source and after most of the processing techniques—is comparable in its physicochemical properties and always biocompatible due to its natural origin [4]. However, it has already been shown that both the donor organism (e.g., allogeneic or xenogeneic sources) and mainly the tissue source (e.g., dermis, pericardium or tendon tissue) have an influence on the integration behavior of collagen-based biomaterials and factors, such as its standing time or its vascularization, which has a major impact on the material functionality in an intended clinical indication [5,6]. Alongside decellularization and sterilization techniques, donor organisms and tissue sources are assumed to be the main factors that have an impact on the physicochemical properties of the resulting biomaterial [7].

Therefore, it was revealed that the specific immunological tissue response to every biomaterial depends on its individual physicochemical properties [8,9]. This can vary from a nearly bioinert response to a strong inflammatory reaction, associated with abscess formation or fibrotic capsule formation, as well as local and systemic consequences [10]. In this context, the role of macrophages has recently been investigated, as these cells are key elements in the tissue reaction cascade [11]. Macrophages are also thought to play an important role in the framework of the tissue reaction to a biomaterial due to their secretion of cytokines [12]. Therefore, macrophages can roughly be divided into two subtypes based on their overall expression profile: the M1 phenotype is pro-inflammatory, has been shown to be especially present in the early healing phase and also seems to be involved in the degradation process of biomaterials inducing a foreign body response, while the M2 phenotype is an anti-inflammatory expressing reparative cytokine and is integrated in the tissue healing phase [13]. Biomaterial-associated multinucleated giant cells (BMGCs) are another cell that may exhibit a pro-inflammatory (M1-BMGCs) or anti-inflammatory (M2-BMGCs) phenotype, which is equivalent to macrophages and dependent on the physical and chemical properties of the biomaterial [14,15,16]. Moreover, it has been shown that these cells are involved in the phagocytic degradation of different biomaterials expressing lytic enzymes [17]. In this context, it has been concluded that these “non-physiological cells” are only involved in material rejections or failures, but the research on this topic has shown that their involvement in the tissue reaction to a specific biomaterial is strongly dependent on the type of biomaterial and its physicochemical properties [13]. Interestingly, it has been assumed that their induction even in the case of collagen-based materials may be contraindicated, but there is still a lack of existing knowledge. Thus, it is highly important to analyze the tissue reaction to a biomaterial for clarification of its specific inflammatory tissue reaction pattern with special consideration of its intended use.

In the context of GBR procedures, it has been understood that barrier membranes should not only provide a barrier function, but they should optimally induce help to modulate the microenvironment to increase bone remodeling [2]. It has been revealed that angiogenesis has been emphasized to be an important factor strongly influencing the outcome of bone healing [18]. It has been discussed that GBR membranes should allow for a so-called “transmembraneous vascularization”, allowing the formation of blood vessels to increase the formation of new bone. However, it has also been shown that an ingrowth of connective tissue is most often necessary to allow the simultaneous ingrowth of blood vessels [19]. This tissue ingrowth has been shown to correlate with the premature breakdown or fragmentation and an associated loss of barrier functionality of collagen membranes [5]. Thus, it is of great importance to develop a next generation of resorbable GBR membranes with an adapted integration behavior that combines both the barrier functionality as well as a transmembraneous blood supply. In this context, it is also of special interest to analyze the overall immune response to a membrane for evaluation of the local events that might additionally support the underlying bone regeneration process.

Furthermore, the alignment of the inflammatory cascade caused by the biomaterial can influence aspects such as the response of anabolic tissue cells, e.g., osteoblasts [9]. Moreover, it has been shown that different interactions between material-induced immune cells (macrophages and/or BMGCs) and tissue cells (osteoblasts or endothelial cells) exist and may support the process of tissue regeneration at the molecular level [20]. During bone healing, interactions between inflammatory response cells and bone remodeling cells have gained more importance [12]. It has been supposed that the next generation of biomaterials should integrate the immune system into regenerative strategies [8,21]. 

Altogether, it is believed that collagen membranes, even for GBR procedures, should provide a long-standing time of several weeks up to 3–4 months for optimal clinical results in the course of jawbone healing. This assumption appears reasonable in the case of multidimensional or bigger bone defects, but it has been reported in the case of “normal” or smaller defects (e.g., in the case of extraction sockets) that the application of native dermis-derived collagen membranes with a very short standing time contributes to comparable bone healing results [19]. In this context, it has been revealed that a fast membrane degradation might also correlate with a higher or more pro-inflammatory alignment of the material-associated tissue reaction [19]. As a consequence, it has been revealed that the faster tissue ingrowth also includes phagocyting cells, such as BMGCs, in concert with macrophages [22]. However, this fast membrane degradation is also combined with a higher transmembraneous vascularization that has been identified as an important co-factor for the material-related bone healing process [6]. Altogether, the question arises concerning which material factors are optimal for combining both the barrier functionality and the support for creation of a molecular microenvironment that triggers the (underlying) process(es) of bone healing. Interestingly, even inflammatory cells, such as BMGCs and macrophages, have been shown to be potent sources of angiogenic molecules, such as the vascular endothelial growth factor (VEGF), so it might be of special interest to create membranes with a higher “inflammatory potential” to create the above-mentioned micromilieu [23].

Particularly in the case of collagen, only a few studies have analyzed the differences in various xenogeneic sources, and scarce knowledge exists about the consequences of the usage of different animal sources as a basis for biomaterials, such as GBR membranes [24,25,26]. Most of the membranes used in GBR procedures are based on porcine donor tissue. Generally, fewer barrier membranes are used that are sourced from other animals or are of synthetic origin [3]. In this context, it has already been demonstrated that collagen membranes derive from pigs, which have been treated using different purification and fabrication processes to induce varying tissue reactions [19]. Some collagen membranes are degraded by mononuclear cells during a very mild tissue reaction producing a long-standing time, while other membranes induce stronger inflammatory reactions, involving BMGCs that lead to a faster degradation combined with a lower standing time or premature breakdown and loss of functionality. Altogether, these differing tissue reactions can be attributed to differences in the preparation processes [7]. Although the influence of different processing methods on collagen-induced tissue reactions has been investigated, the influence of the collagen animal source is rarely analyzed. To fill this knowledge gap, the inflammatory tissue reactions to two collagen membranes from porcine and bovine origins were analyzed in the present study. The membranes were implanted over a period of up to 60 days by means of an established preclinical implantation model in Wistar rats. Furthermore, established histopathological and histomorphometrical analysis methods, as well as immunohistochemical detection methods of M1- and M2-macrophages, were applied [14,27].

## 2. Materials and Methods 

### 2.1. Bovine Collagen Membrane 

The collagen membrane analyzed in the present study is based on a native collagen obtained from bovine skin. The collagen within the structure of the membrane is mostly composed of collagen Type I fibers. A hydrogel was prepared from the precursor tissue and then freeze-dried to create the membranes. The membranes were not chemically cross-linked.

### 2.2. Porcine Collagen Membrane

The porcine collagen membrane analyzed in the present study is based on native collagen derived from pericardium using a decellularization process including a wet-chemical treatment, lyophilization and final sterilization by ethylene oxide gas. The membrane was shown to fulfill the requirements of EN ISO 10993-1 and EN ISO 7405.

### 2.3. In Vivo Study

The Local Ethical Committee of the Faculty of Medicine (University of Niš, Serbia) authorized the in vivo experiments prior to the biomaterial implantations, based on decision number 323-07-09101, on 2020-05/5, of the Veterinary Directorate of the Ministry of Agriculture, Forestry and Water Management of the Republic of Serbia. 

The preclinical in vivo experiments were conducted at the Faculty of Medicine in the University of Niš (Serbia). Animal housing was conducted using standard conditions, i.e., water ad libitum, artificial light and regular rat pellets, as well as standard pre- and postoperative care. 

**Figure 1:**
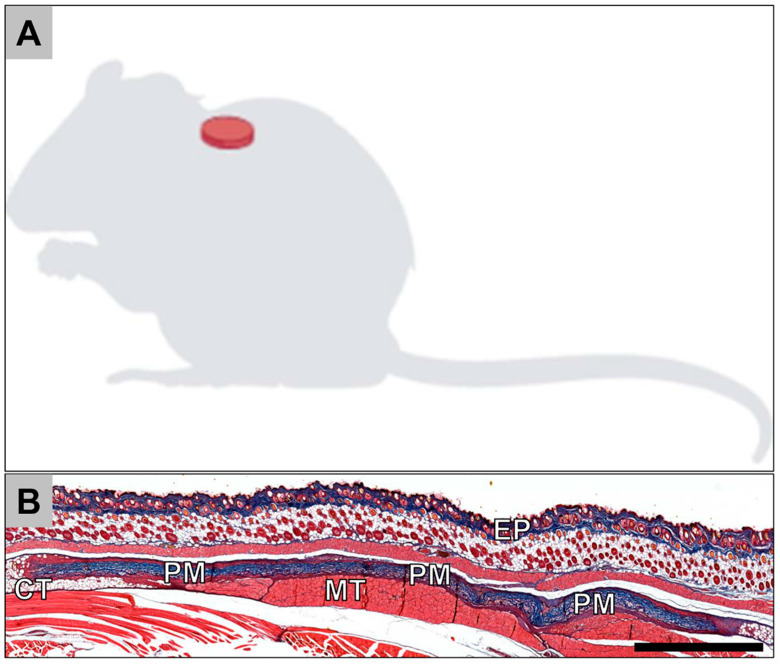
(**A**) Schematic image of the implantation site. (**B**) Overview of a subcutaneous implantation site of the porcine collagen membrane (PM) at day 30 postimplantation. CT = connective tissue; EP = epidermis (Azan-staining, “total scan”, 100× magnification, scalebar = 1 mm).

In total, 30 female, 10–12-week-old Wistar rats that were obtained from the Military Medical Academy (Belgrade, Serbia) were randomly allocated into two study groups. Each of the two study groups contained 15 experimental animals; 5 animals were sacrificed for each group per time point (n = 5), i.e., 10, 30 and 60 days. The implantation was conducted following the protocol described by Barbeck et al. [27,28,29,30,31,32]. In brief, the animals were anesthetized via an intraperitoneal injection (10 mL ketamine [50 mg/mL] with 1.6 mL Xylazine [2%]). After shaving and disinfection, an incision down to the subcutaneous tissue within the rostral subscapular region was made. Subsequently, a subcutaneous pocket was bluntly built by a scissor, and the biomaterials were implanted into the pocket (Figure 1). Afterwards, the wounds were sutured. 

After the respective study time points, the animals were euthanized with an overdose of the above-mentioned anesthetics, and the implantation area together with the surrounding tissue was explanted. Subsequently, the explanted tissue was fixed using a 4% formalin solution for 24 h and then placed into PBS for the following histological workup process.

#### 2.3.1. Histology and Immunohistochemistry

For histological workup, the tissue explants were initially cut into two segments of identical dimensions and dehydrated using a series of increasing alcohol concentrations. After a xylol exposure, paraffin embedding was performed. Sections were prepared with a thickness of 3–5 μm by means of a rotation microtome (SLEE, Mainz, Germany). Three sections of every tissue explant were used for histochemical staining, i.e., hematoxylin and eosin (H&E), Movat Pentachrome and Azan.

Furthermore, four additional sections of every tissue explants were used for immunohistochemical detection of macrophages. NF kappa ß-positive M1 macrophages and CD163-postive M2 macrophages were stained by means of antibodies against the pro- and anti-inflammatory molecules based on previously published methods [15]. Briefly, the slides were initially treated with citrate buffer and proteinase K in a water bath for 20 min that had a temperature of 96 °C and a pH 8. This was followed by equilibration using TBS-T buffer. Subsequently, the slides were prepared by H_2_O_2_ and avidin and biotin blocking solutions (Avidin/Biotin Blocking Kit, Vector Laboratories, Burlingame, CA, USA), incubated with the respective first antibody for 30 min, followed by incubation with the secondary antibody (goat anti-rabbit IgG-B, sc-2040, 1:200, Santa Cruz Biotechnology, Dallas, TX, USA). Afterwards, the avidin–biotin–peroxidase complex (ThermoFisher Scientific, Henningsdorf, Germany) (30 min) was applied, and a counterstaining by hemalum was conducted. 

#### 2.3.2. Histological Analysis 

The histological analyses to study the tissue–biomaterial interactions within the implantation beds of the biomaterials and their surrounding tissue were conducted using an Axio Imager M2 (Zeiss, Oberkochen, Germany) based on a protocol according to the DIN ISO 10993-6 as previously described [11,23,33,34,35]. These analyses focused on the evaluation of the following parameters within the framework of the early and the late tissue response related to the implants: fibrosis; hemorrhage; necrosis; vascularization; and the presence of neutrophils, lymphocytes, plasma cells, macrophages and biomaterial-associated multinucleated giant cells (BMGCs). Finally, microphotographs were taken with an Axiocam 506 color connected to a computer system running the ZEN Core (Zeiss, Oberkochen, Germany) connected to a microscope. 

#### 2.3.3. Histomorphometrical Analysis 

The histomorphometrical analyses included the measurements of the occurrence of anti-inflammatory and pro-inflammatory cells within the implant beds of the membranes as previously described [36]. Briefly, the slides stained by the aforementioned immunohistochemical methods were initially digitized. Then, the Image J software (National Institutes of Health, Bethesda, MD, USA) enabled the measurements of the stained cells within the total scans. At first, the defect area and the membrane area were manually marked, and their areas were determined. After that, the number of macrophages was also measured via a specially programmed plugin that allowed us to mark the area of the red stained cells automatically [37]. Finally, the cell numbers were related to the respective total area to calculate the numbers of cells per mm^2^ (macrophages/mm^2^).

#### 2.3.4. Statistical Analysis 

Quantitative data are shown as mean ± standard deviation after an analysis of variance (ANOVA), which enabled comparison of the data from the study groups via the GraphPad Prism 8.0 software (GraphPad Software Inc., La Jolla, CA, USA). Statistical differences were designated as significant if *p*-values were less than 0.05 (* *p* ≤ 0.05), and highly significant if *p*-values were less than 0.01 (** *p* ≤ 0.01), less than 0.001 (*** *p* ≤ 0.001) or less than 0.0001 (**** *p* ≤ 0.0001).

## 3. Results

### 3.1. Histological (Qualitative) Analysis

**Figure 2:**
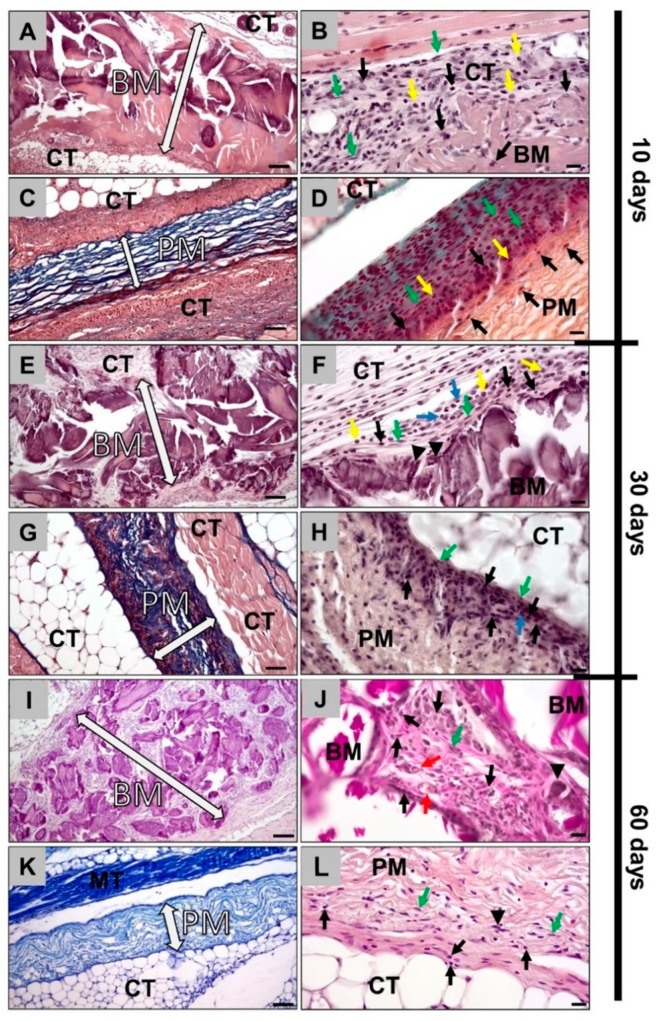
Histopathological images of bovine and porcine collagen membranes at days 10, 30 and 60. (**A**) Bovine collagen membrane at day 10 (hematoxylin and eosin (HE) staining, magnification = 200×, scale bar = 50 µm). (**B**) Bovine collagen membrane at day 10 (hematoxylin and eosin (HE) staining, magnification = 400×, scale bar = 20 µm). (**C**) Porcine collagen membrane at day 10 (Azan staining, magnification = 200×, scale bar = 50 µm). (**D**) Porcine collagen membrane at day 10 (Movat’s Pentachrome staining, magnification = 400×, scale bar = 20 µm). (**E**) Bovine collagen membrane at day 30 (hematoxylin and eosin (HE) staining, magnification = 200×, scale bar: 50 µm). (**F**) Bovine collagen membrane at day 30 (hematoxylin and eosin (HE) staining, magnification = 400×, scale bar = 20 µm). (**G**) Porcine collagen membrane at day 30 (Azan staining, magnification = 200×, scale bar = 50 µm). (**H**) Porcine collagen membrane at day 30 (hematoxylin and eosin (HE) staining, magnification = 400×, scale bar = 20 µm). (**I**) Bovine collagen membrane at day 60 (hematoxylin and eosin (HE) staining, magnification = 200×, scale bar = 50 µm). (**J**) Bovine collagen membrane at day 60 (hematoxylin and eosin (HE) staining, magnification = 400×, scale bar= 20 µm). (**K**) Porcine collagen membrane at day 60 (Giemsa staining, magnification = 200×, scale bar= 50 µm). (**L**) Porcine collagen membrane at day 60 (hematoxylin and eosin (HE) staining, magnification = 400×, scale bar = 20 µm). BM: bovine membrane; PM: porcine membrane; MT: muscle tissue; CT: connective tissue; white arrow: borders of the membrane; black arrows: macrophages; green arrows: fibroblasts; yellow arrows: eosinophils; blue arrows: neutrophils; black arrowheads: multinucleated giant cells; red arrows: blood vessels.

Histopathological analysis of the bovine collagen membrane at 10 days after implantation showed that the membrane was intact, showing no signs of a breakdown or fragmentation (Figure 1A). At the material surfaces, signs of a minor inflammatory tissue reaction were detected (Figure 2B). The reactive tissue was mostly composed of macrophages, granulocytes and fibroblasts at the surfaces and surrounding the membrane (Figure 2B). As for the porcine membrane, similar observations were noted at day 10 post implantation (Figure 2C,D).

At 30 days post implantation, the first signs of tissue infiltration were found in the case of the bovine collagen membranes (Figure 1E). Therefore, an increased intensity of the reactive tissue could be detected (Figure 2E). The tissue was mainly composed of the same cell types found at 10 days post implantation. Thus, mainly macrophages were found at the material surfaces. Moreover, single biomaterial-induced multinucleated giant cells (BMGCs) were found at the surface of the bovine membrane at this time point (Figure 2F). Similar observations were made in the group of the porcine collagen membrane that was found to be completely intact at this time point (Figure 2G). Thus, a layer of macrophages was found attached to the membrane surfaces (Figure 2G). Nonetheless, no BMGCs appeared to be attaching to the porcine membrane (Figure 2H).

At day 60 post implantation, the bovine membrane appeared to be fragmented, as big fragments of the membrane were found to be overlapping within the subcutaneous connective tissue (Figure 2I). Furthermore, the reactive tissue infiltrated the interspaces of the membrane fragments (Figure 2I). Similar cell types to those observed at days 10 and 30 were found within the surrounding tissue, i.e., mainly macrophages in concert with single BMGCs (Figure 2J). Additionally, single vessels were observable within the tissue that infiltrated this membrane type (Figure 2J). The porcine membrane appeared intact with less intense reactive tissue response and infiltration (Figure 2K). Attached to the porcine membrane, mainly macrophages and fibroblasts but also a few BMGCs appeared, but no transmembraneous vascularization was observed (Figure 2L).

**Figure 3:**
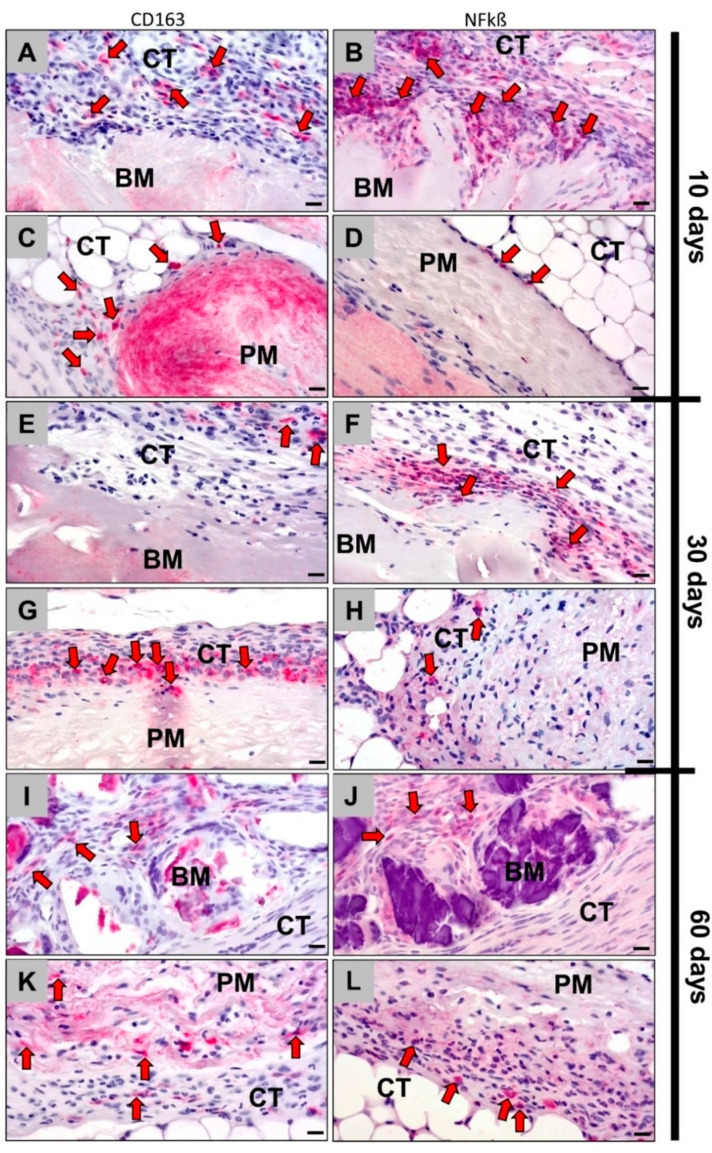
Immunohistochemically stained slides show detection of CD163-positive M2 macrophages (left column: **A**,**C**,**E**,**G**,**I**,**K**) and NF-kß-positive M1 macrophages (right column: **B**,**D**,**F**,**H**,**J**,**L**) into the implantation beds of both bovine and porcine collagen membranes at days 10, 30 and day 60 after implantation (all images: 400× magnification; scale bars = 20 μm) (left: CD163 immunohistochemical staining; right: NF-kß immunohistochemical staining). BM: bovine membrane; PM: porcine membrane; CT: connective tissue; red arrows: CD163- and NF-kß-positive macrophages.

Within the implantation sites of bovine collagen membranes at 10 days after implantation, the immunohistochemical detection showed that more NF-kß-positive M1 macrophages were present compared to the number of CD163-postive M2 macrophages (Figure 3A,B). Additionally, visibly more M1 macrophages than CD163-positive M2 macrophages appeared at the surface of the porcine membrane, but the numbers of immunohistochemically stained cells were lower in the case of this membrane type (Figure 3C,D).

At day 30 post implantation, still more NF-kß-positive M1 macrophages than M2 macrophages appeared on the surface of both membranes (Figure 3E,F). In contrast to day 10, comparable amounts of CD136-positive M2 presence were found in both groups, while the numbers of the M1 macrophages were visibly higher in the group of the bovine membrane (Figure 3G,H). 

At day 60 post implantation, both collagen membranes induced comparably lower numbers of M1 macrophages than at day 30 (Figure 3I–L). However, the numbers of M2 macrophages were found to be comparably low in the group of the bovine membrane, while the M2 macrophage number increased in the group of the porcine membrane (Figure 3I–L). 

**Figure 4:**
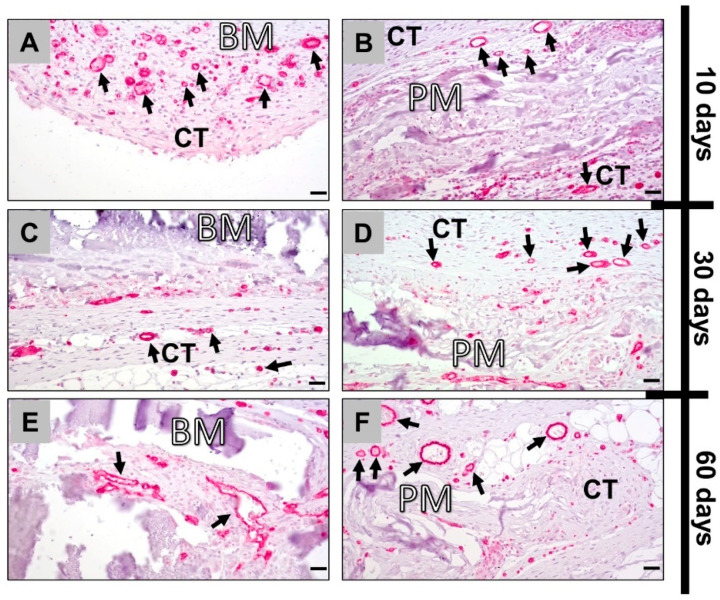
Immunohistochemically stained slides show detection of CD31-positive endothelial cells into the implantation beds of both bovine (left column: **A**,**C**,**E**) and porcine collagen membranes (right column: **B**,**D**,**F**) at days 10, 30 and 60 after implantation (all images: 400× magnification; scalebars = 20 μm). CT: connective tissue; BM: bovine membrane; PM: porcine membrane; black arrows: CD31-positive vessels.

The immunohistochemical detection of blood vessels showed an elevated vascularization of the surrounding tissue around the bovine membrane comparative to the porcine membrane at day 10 post implantation (Figure 4A,B). At day 30 post implantation, both collagen membranes exhibited a comparable vascularization within the connective tissue surrounding the implants and at the tissue–membrane interface (Figure 4C,D). At 60 days postimplantation, vessels were found within the tissue that infiltrated the bovine membrane leading to a transmembraneous vascularization, while only single small blood vessels were detected within the material bodies of the porcine collagen membrane (Figure 4E,F). 

### 3.2. Histomorphometrical (Quantitative) Analysis

**Figure 5:**
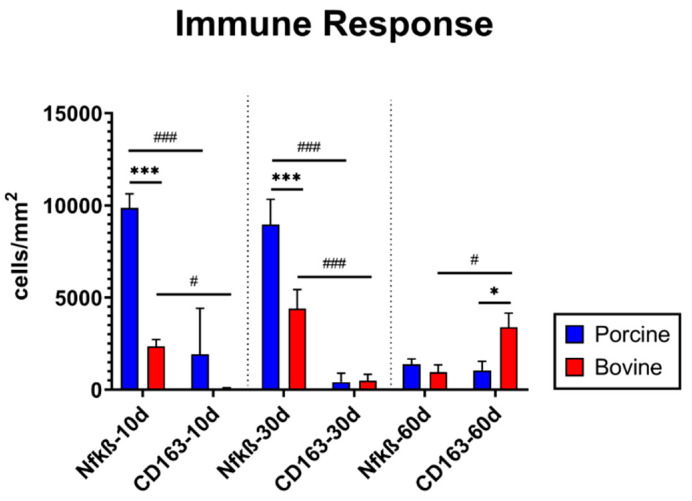
Results of the histomorphometrical analysis of the immune response within the implantation beds of both materials (*/# *p* ≤ 0.05, ***/### *p* ≤ 0.0001).

The quantitative analysis of pro- and anti-inflammatory cells showed that there were comparable numbers of CD163-positive M2 macrophages found in the implantation beds of the bovine collagen membrane (71.6 ± 42.2 cells/mm^2^) and porcine collagen membrane (1925.4 ± 2489.2 cells/mm^2^) at day 10 after implantation (Figure 5). In contrast, significantly more NF-kß-positive M1 macrophages were detected in the samples of the bovine collagen membrane (2352.8 ± 369.1 cells/mm^2^) compared to the group of the porcine collagen membrane (9862.4 ± 759.6 cells/mm^2^) (*** *p* ≤ 0.001). Furthermore, significantly more M1 macrophages than M2 macrophages (# *p* < 0.05 and ### *p* < 0.001) were found in both study groups at this early time point (Figure 5). 

At 30 days after implantation, comparable values of M2 macrophages were also found in the implantation sites of the bovine collagen membrane (490.2 ± 339.2 cells/mm^2^) and the porcine collagen membrane (413.6 ± 481.5 cells/mm^2^) (Figure 4). Still higher numbers of M1 macrophages were detected in the group of the bovine collagen compared to the value in the group of the porcine membrane (bovine collagen membrane: 4403.0 ± 1031.9 cells/mm^2^; porcine collagen membrane 8954.2 ± 1371.5 cells/mm^2^) (Figure 5). Furthermore, significantly higher numbers of M1 macrophages were found within the implant site of both collagen membranes compared to the M2 subforms (### *p* < 0.001). 

At 60 days after implantation, comparable numbers of NF-kß-positive M1 macrophages were detectable in the implantation bed of the bovine collagen membrane (652.1 ± 391.4 cells/mm^2^) and of the porcine membrane (1380.8 ± 281.7 cells/mm^2^) (Figure 4). At this time point, the bovine membrane induced significantly higher numbers of CD163-positive M2 macrophages (3384 ± 776.3 cells/mm^2^) compared to the numbers of M1 macrophages (# *p* < 0.05) but also compared to the numbers of M2 macrophages in the group of the porcine membrane (1043.4 ± 492.3 cells/mm^2^) (* *p* ≤ 0.05) (Figure 5). 

## 4. Discussion

The concept of an “induced” membrane has already been described in traumatology as a strategy for bone regeneration, particularly in the cases of large bone defects [38]. This method involves a two-stage procedure, where a “biological” membrane is induced as a foreign body response after application at the first stage, acting as a “chamber” for the insertion of autologous bone-graft at the second stage. It has been shown that this induced membrane can possess osteoinductive, osteogenic and angiogenic properties, and several clinical studies have demonstrated satisfactory results [38]. Especially implant bed vascularization via angiogenesis has been recognized as a basic factor for successful (bone) tissue regeneration [39,40]. Thus, to date, different strategies have focused on the development of materials that can promote vascularization. Another goal for the future development of a variety of biomaterials is to induce a specific immune response that can further stimulate (bone) tissue healing [12]. In this context, the inflammatory tissue reaction cascade to biomaterials has been elucidated in recent decades, and especially the macrophage has been identified as an important key factor to guide the tissue regeneration process [14,40]. In broad terms, there are two main subforms of macrophages, i.e., pro-inflammatory M1- and anti-inflammatory M2-macrophages [41]. It is assumed that even the induction of the latter subform by a biomaterial is preferable to support the material-mediated healing process [40]. Moreover, it has been shown that different types of (oral) mesenchymal stem cells (MSCs), such as dental-derived mesenchymal stem cells (D-dMSCs), are involved in (bone) tissue healing [42,43]. Interestingly, it has been revealed that MSCs from oral tissues are highly committed to differentiate toward osteoblasts and precursors of bone tissue, but they also provide immunomodulatory activity [44,45].

Recently, it has also been concluded that the next generation of barrier membranes for Guided Bone Regeneration (GBR) in dentistry and maxillofacial surgery should not only resume a “passive” role in tissue separation [46]. In this context, the membrane should fulfill both functions, i.e., to separate the “soft tissue” and the bone defect site and to contribute to bone tissue regeneration [38,46]. This means that it is expected that a barrier membrane should actively contribute to molecular processes of bone tissue regeneration, such as stem cell differentiation osteoblast ingrowth, or via underlying processes, such as defect site nutrition, i.e., nutrition of the underlying bone tissue defect, and there are only two ways to ensure this functionality: either via diffusion or transmembraneous vascularization [39,43,47]. Together with this functionality, this material type has to fulfill its main objective, that is, to act as a barrier. In this context, the barrier functionality is needed for a minimum of 4–6 weeks for periodontal tissues and 16–24 weeks for bone tissue regeneration [46]. To date, the question remains as to how such a new membrane can be developed.

It is known that native (porcine) dermis-derived collagen membranes are most often prematurely resorbed in 4–8 weeks, but it has been shown that they are “optimally” degraded via more or less physiological processes providing a good biocompatibility [19]. However, it has been reported that they do not undergo a transmembraneous vascularization, which may not be necessary, as their low thickness might allow for a diffusion-based transport of nutrients [19]. To increase the standing time, membranes from other tissues, such as the pericardium, have been used to prepare barrier membranes [6]. It has been shown that such a membrane has a better degradation behavior but also does not undergo significant vascularization due to its lower thickness, which is attributable to the tissue origin. However, the choice of this tissue source is based on the knowledge of the different natural collagen cross-linking, which seems to increase the standing time [6].

In contrast, chemical cross-linking has been manifoldly shown to decrease the biocompatibility of such biomaterials, leading to exaggerated inflammatory tissue responses and premature material breakdown [48]. However, these studies have resulted in very important fundamental findings: The transmembraneous vascularization including the vessel formation within a membrane to bridge especially longer distances, as in the case of thicker barrier membranes, only seems to be possible based on the ingrowth of complex tissue into the material body [48]. However, to trigger tissue ingrowth, the membrane needs to be (partially) resorbed or phagocytosed, which requires the involvement of at least macrophages or multinucleated giant cells (MNGCs) [14]. These cells are then also involved in angiogenic processes due to their expression of respective molecules, such as the vascular endothelial growth factor (VEGF) [14]. Thus, it can be concluded that it might be necessary to create membranes with a higher “inflammatory potential” to create the above-mentioned micromilieu [23].

Particularly in the case of collagen-based barrier membranes, only a few studies have analyzed the differences in various xenogeneic sources, and scarce knowledge exists about the consequences of the usage of different animal sources [24,25,26]. Most of the membranes used in GBR procedures are based on porcine donor tissue. Generally, fewer barrier membranes are used that are sourced from other animals [3]. In this context, it has already been demonstrated that collagen membranes derived from pigs induce varying tissue reactions [19]. Interestingly, most of these differing tissue reactions can be attributed to differences in the preparation processes [7]. Although the influence of different processing methods on collagen-induced tissue reactions has been investigated, the influence of the collagen animal source is rarely analyzed. The properties of collagen barriers may also be affected by the origin of the collagen [49,50]. Cross-linking happens physiologically in native tissue; therefore, different animal and tissue sources can provide different cross-linking degrees that might be an alternative to adverse chemical cross-linking. However, only poor knowledge exists about this topic.

To fill this knowledge gap, the inflammatory tissue reactions to a collagen membrane from bovine dermis were analyzed in the present study. This is of special interest, as it has been reported that this origin tissue occupies higher pre-existing cross-links in the collagen [48]. Thereby, this newly developed barrier membrane based on porcine collagen was compared to a manifoldly studied and commercially available pericardium membrane with a well-described resorption and cell reaction profile [51]. The characteristics of both origin tissues are listed in Table 1.

Both membranes were implanted for up to 60 days using the subcutaneous implantation model in Wistar rats. Furthermore, established histopathological, immunohistochemical detection methods of M1- and M2-macrophages and blood vessels and histomorphometrical analysis methods were applied [14,27].

The results of the histopathological analyses of the tissue responses to the membranes and their integration behavior showed that the bovine collagen membrane was intact, showing no signs of a breakdown or fragmentation at day 10 post implantation. Starting with day 30, the first signs of tissue infiltration were found, and at day 60 post implantation, the bovine membrane was fragmented. However, the fragments of the membrane were found to be overlapping within the subcutaneous connective tissue. Therefore, this membrane type induced a tissue reaction, including both macrophages and multinucleated giant cells (MNGCs), that promoted this fragmentation process. Interestingly, the reactive tissue infiltrated the interspaces of the membrane fragments, and high numbers of vessels were found within the tissue that infiltrated the membrane type. In contrast, the porcine membrane remained intact until day 60 post implantation, inducing a less intense reactive tissue response and only a single cell infiltration of mononuclear cells. Furthermore, no transmembraneous vascularization was detected, but some single small vessels were found within the membrane.

Altogether, this analysis part revealed clear differences in the inflammatory tissue response and the integration behavior but also in the vascularization pattern. Therefore, the bovine membrane underwent fragmentation mediated by phagocyting cells, i.e., macrophages and MNGCs, as already shown in other publications about different collagen membranes from other sources [22,54]. Thus, the results could substantiate different former study results. However, the integration and the fragmentation pattern of this membrane is unique, as the membrane was not completely fragmented into two or more parts, leading to a direct contact between the overlying and underlying tissue (compartments). In contrast, this membrane type disintegrated into smaller subunits that were further surrounded by a cell and especially vessel-rich tissue. Moreover, the subunits were found to be overlapping within the tissue, which leads to the conclusion that the membrane still seems to prevent the invasion of tissue from “one compartment into the other”. It is thus conceivable that this membrane will still maintain its barrier functionality. This special integration and degradation pattern has never been described for other membranes and can be defined as “secondary porosity”, which makes the analyzed bovine membrane the first of a new generation. In contrast, the porcine collagen membrane induced a tissue reaction and an integration behavior that has manifoldly been described as a remodeling and incorporation without any signs of fragmentation [51]. 

In this context, the collagen type and its cross-linking degree due to the different tissue appear to be the reasons for this integration behavior of the bovine collagen membrane. Its compactness may have led to the observed inflammatory tissue response. Therefore, the observed inflammatory tissue response mediated the integration pattern.

Additionally, the study results regarding the observation of the inflammation-driven transmembraneous vascularization reaffirmed former study results published by Ghanaati and colleagues that described the VEGF expression of MNGCs and the dependence of the implant bed vascularization, especially of bone substitute materials, but also of collagen-based materials on the occurrence of this multinucleated cell type [19,23]. This observation leads to the conclusion that the analyzed vascularization pattern of the bovine membrane is connected to the material-induced inflammatory tissue response, which also makes this membrane type a first prototype of an immune-modulating material. Thus, the control over the integration pattern and the material-induced inflammation may be an alternative to conventional membranes.

Finally, the histomorphometrically measured occurrence of M1- and M2-macrophages revealed increased occurrences of pro-inflammatory cells induced by the porcine membrane at 10 and 30 days in opposite to the bovine group. However, their occurrence was strictly local and did not affect the surrounding tissue. Furthermore, a decrease at day 60 to comparable numbers compared to the group of the porcine membranes was found. Thus, it is conceivable that the initially increased numbers of pro-inflammatory cells might be induced by the differently cross-linked membrane initiating the fragmentation process. However, the observed declined levels of M1 and M2 macrophages at day 60 post implantation that showed comparable levels of both subforms suggest that both membranes do not induce a chronic inflammation that can cause inflammatory-driven implant failures. Thus, both biomaterials seem to be fully biocompatible. Interestingly, the porcine group showed significantly increased M2 macrophage levels at day 60 compared to the M1 macrophages numbers. This might indicate that this membrane can integrate within the implantation bed with molecular support of the tissue healing process. 

To summarize, the importance of the bioactivity of the membrane has recently been emphasized to create a so-called “bioactive membrane compartment”, which means to setup an underlying micromilieu suitable for bone tissue regeneration [2]. The bovine collagen membranes seem to allow for the establishment of such a micromilieu due to its special degradation and integration pattern that triggers transmembraneous vascularization. The bovine collagen membranes seem to allow for the establishment of such a micromilieu due to its special degradation and integration pattern that triggers transmembraneous vascularization. In this context, the vascularization pattern allows the creation of local differences in oxygenation and presumably the availability of nutrients that might also help to generate an appropriate niche environment for osteoprogenitors [55]. Thus, these findings might be also relevant in the context of bone repair, which involves blood vessel growth and pro-angiogenic signaling interactions. Furthermore, the nutrition of the defect site may support the survival of cells implanted with the membrane, such as different oral-derived stem cells or (pre-) osteoblasts in tissue engineering applications [42,43].

Additionally, the membrane subunits were found to be overlapping within the tissue, which leads to the conclusion that the membrane still maintains its barrier functionality. This special integration and degradation pattern can be defined as “secondary porosity”, which makes the analyzed bovine membrane the first of a new generation that modulates the immune response to support bone defect healing. 

The present results lead to the conclusion that this membrane meets the requirements for a GBR barrier membrane with an enhanced standing time that seems optimal and does not trigger fast degradation like in some cases of artificially cross-linked collagen [48]. However, the age and region of the bovine sources have shown discrepancies in the degree of cross-linking, which can cause a batch-to-batch manufacturing inconsistency. A standardization of the process of animal domestication, collagen extraction and collagen reconstruction can solve this problem. Nonetheless, bovine collagen, extracted from the skin, can provide an optimized standing time for different clinical indications. 

## 5. Conclusions 

The results of the present in vivo study showed that dermis-extracted bovine collagen membranes underwent a special integration behavior by providing a “secondary porosity” in concert with a transmembraneous vascularization that is expected to be suitable for Guided Bone Regeneration applications. Histological analysis showed comparable results to the pericardium-extracted porcine-sourced collagen membranes. The bovine membrane remained intact initially, and the delayed fragmentation was accompanied with granulation tissue infiltration and the appearance of multinucleated giant cells that seemed to mediate transmembraneous vascularization. This vascularization can serve as an advantage in GBR alongside the vascularization of the implant bed, suggesting a further functionality of barrier membranes that current commercially available materials do not provide. Furthermore, the delayed fragmentation of the bovine membrane suggests a longer standing time, which can be beneficial for certain dental or maxillofacial indications where conventional resorbable barrier membranes suffer from a short lifetime. Altogether, the analyzed bovine membrane might be an alternative to artificially cross-linking membranes, as such processing can cause bioincompatibility and premature resorption that would result implant failure.

## Figures and Tables

**Table 1:** Comparison between collagen-based barrier membranes from different xenogeneic resources and extraction sites for Guided Bone Regeneration (GBR).

	Porcine		Bovine	
**Common tissue source**	Dermis	Compactness of tissue hinders cell infiltration and degradation in vivo [48].	Dermis	Compactness of tissue hinders cell infiltration and degradation in vivo [48].
	Pericardium	Lower tissue reaction and higher cell infiltration in vivo than dermis-derived collagen [6].	Achilles tendon	Balanced tissue reaction and adequate cell infiltration [5].
**Collagen types**	I, III		I, II, IV [52]	
**Use in GBR**	Common		Not as common	
**Immunogenicity**	Lower [53]		Higher (3% of population is allergic) [53]	
**Religious limitations**	In Islam		In Hinduism	
